# Improvement of Epitope Prediction Using Peptide Sequence Descriptors and Machine Learning

**DOI:** 10.3390/ijms20184362

**Published:** 2019-09-05

**Authors:** Cristian R. Munteanu, Marcos Gestal, Yunuen G. Martínez-Acevedo, Nieves Pedreira, Alejandro Pazos, Julián Dorado

**Affiliations:** 1 RNASA-IMEDIR, Computer Science Faculty, University of A Coruna, 15071 A Coruña, Spain; 2 Biomedical Research Institute of A Coruña (INIBIC), University Hospital Complex of A Coruña (CHUAC), 15006 A Coruña, Spain; 3 Centro de Investigación en Tecnologías de la Información y las Comunicaciones (CITIC), Campus de Elviña s/n, 15071 A Coruña, Spain; 4 Unidad Profesional Interdisciplinaria de Biotecnología, National Polytechnic Institute (IPN), Ticoman, 07340 Mexico City, Mexico

**Keywords:** epitopes, machine learning, protein sequences, qualitative structure–activity relationships

## Abstract

In this work, we improved a previous model used for the prediction of proteomes as new B-cell epitopes in vaccine design. The predicted epitope activity of a queried peptide is based on its sequence, a known reference epitope sequence under specific experimental conditions. The peptide sequences were transformed into molecular descriptors of sequence recurrence networks and were mixed under experimental conditions. The new models were generated using 709,100 instances of pair descriptors for query and reference peptide sequences. Using perturbations of the initial descriptors under sequence or assay conditions, 10 transformed features were used as inputs for seven Machine Learning methods. The best model was obtained with random forest classifiers with an Area Under the Receiver Operating Characteristics (AUROC) of 0.981 ± 0.0005 for the external validation series (five-fold cross-validation). The database included information about 83,683 peptides sequences, 1448 epitope organisms, 323 host organisms, 15 types of in vivo processes, 28 experimental techniques, and 505 adjuvant additives. The current model could improve the in silico predictions of epitopes for vaccine design. The script and results are available as a free repository.

## 1. Introduction

The term proteome was used for the first time in 1994 and was defined as the total proteins expressed by a genome in a tissue or in a cell. It is said that its main characteristic is to be dynamic, due to its components, as proteins vary depending on the tissue, cell, or cell compartment, and these can change in response to their microenvironment—for instance stress, temperature, drug action, etc. Proteomics unlocks the paths to the search for clinically useful biomarkers of diseases, treatment response, and aging. Currently, proteomics research has application in different fields of science and industry, so a large amount of information contained in public databases is available [1]. In addition, the fast growth of bioinformatics techniques and their applications, in conjunction with the substantial quantity of experimental information, have created a big impact on immunology analysis.

Vaccination is one of the most effective techniques to prevent infectious diseases. Upon vaccination, a foreign antigen interacts with the host immune system, which evokes an immune response. The immunizing agent could also be an intact inactivated version of the pathogens or parts of the pathogens that are able to generate a strong immunogenic response. The understanding of the antigen molecular interactions resulted in the development of improved peptide vaccines. Peptide vaccines consist in the discovery and synthesis of B-cell and T-cell epitopes. These peptides are able to induce a specific immune response. Epitopes are recognized by the immune system parts as antibodies, B cells, and T-cells [2,3,4]. The epitope fragments could be found in foreign and self-proteins. They can be categorized depending on their structure and how they integrate with the paratope [5]. T-cell epitopes need a bond with the major histocompatibility (MHC) molecules in order to induce an immune response, and they can be found on the surface of an antigen-presenting cell. The key role in the immune system is represented by the recognition of the epitopes by the T-cells. Small variations of the normal immunity function could have a grave impact on the organism. In specific cases of autoimmune diseases, the peptides of the native cells are miss-recognized by the T-cells as foreign peptides. Thus, native peptide could be attacked, resulting in a modification of the normal tissues [6].

A possible method to find new T-cell epitopes is the prediction of MHC binding needed for the T-cell recognition [7]. This is extremely difficult to be put into practice by experimental techniques using an extensive set of alleles. Thus, the bioinformatics solutions are needed for developing prediction methods of epitopes. Data-driven solutions are the most successful for the prediction of T-cell epitopes. The T-cell epitope in silico prediction is usually based on previous information such as the peptide-binding specificity to MHC alleles [8]. Different prediction algorithms for T-cell epitopes have been constructed using peptide sequence and experimental affinity. The Immune Epitope Database (IEDB—http://www.iedb.org) [9] is a freely available resource that classifies experimental data on immune epitopes (the molecular targets of adaptive immune responses) studied in humans, non-human primates, and other animal species in the context of infectious diseases, allergy, autoimmunity, and transplantation, with the aim of keeping the findings of recent research available and updated.

The computational techniques are aimed at finding mathematical relationships in the form of equations between datasets that have been obtained based on experimental work or have been calculated using theoretical considerations. Studies of correlations between physical properties and chemical or biological properties require quality biological data, define relevant chemical descriptors, and choose a suitable model to predict the biological function of peptides. The molecular information about a peptide could be its 3D structure or its simple amino acid sequence. The computational methods to find a mathematical model to predict peptide function using its molecular information are numerous, from linear to non-linear algorithms. The qualitative structure–activity/property relationships (QSAR/QSPR) [10,11] represent very useful methods that are able to predict molecular activity or property using molecular descriptors. In order to find the mathematical function/algorithm that is able to map the inputs (molecule descriptors/characteristics) to the output (molecule biological activity), linear and non-linear Machine Learning (ML) methods are used. In order to encode the changes on B-cell epitope activity after multiple variations/perturbations in factors such as the sequences of the peptides, host organism, source organism, immunological process, and experimental technique, the perturbation theory (PT) is introduced to mix the original molecular descriptors with these factors [12,13]. This feature transformation through mixing information is an alternative to the classical ML one-hot representation of the categorical features (binary features for each category).

Different groups developed ML models to predict B-cell epitope activity after multiple variations/perturbations in experimental conditions. Gonzalez-Diaz et al. [14] developed the first linear model for B-epitope prediction using 200,000 cases of perturbations such as structural changes in peptides from assay involving 500 source organisms, 50 host organisms, 10 biological processes, and 30 experimental techniques. The reported accuracy, sensitivity, and specificity were >90% (training and validation series). Vázquez-Prieto et al. [15] compared the PT-ML models for B-cell epitopes prediction using different physicochemical molecular properties of peptide sequences. All these models do not include important factors such as adjuvant additives in vaccine design. In addition, the datasets of the studies were based on a previous version of IEDB.

Martinez-Arzate et al. presented the latest published model for epitopes [16]. This model was based on Shannon’s information as peptide molecular descriptors and experimental condition perturbations. Thus, the dataset was based on 1,048,190 pairs of query and reference peptide sequences and perturbations in sequence or assay conditions: 1448 epitope organisms, 323 host organisms, 15 types of in vivo processes, 28 experimental techniques, and 505 adjuvant additives. This linear model was characterized by modest values of accuracy, sensitivity, and specificity (71–80%). Therefore, these results were improved by the current study using non-linear ML methods, better metrics for unbalanced datasets (area under the receiver operating characteristics—AUROC [17]), reproducible open-access python scripts, and multiple dataset splits (n-fold cross-validation) for statistical significance of the results.

## 2. Results

The main Jupyter notebook uses pipelines from sklearn (python) [18], standardization scaling of data (standard deviation units), five-fold cross-validation (outer validation using stratified folds for unbalanced classes), and seven Machine Learning methods, such as k-nearest neighbors algorithm (KNN) [19], support vector machine (SVM linear and SVM non-linear based on radial basis functions, RBF) [20], logistics regression (LR) [21], decision tree (DT) [22], random forest (RF) [23], and XGBoost—an optimized distributed gradient boosting library (XGB) [24]. The performance of the models was characterized applying the Area Under the Receiver Operating Characteristics (AUROC) [17]. In order to be able to reproduce the project results, all the scripts, datasets and results are available as a GitLab repository (https://gitlab.com/muntisa/machine-learning-for-peptide-epitopes/).

The AUROC values for the used ML methods are shown in Table 1. The best classifier using the default parameters of all the ML methods and class weights for unbalanced classes was provided by random forest (10 estimators/decision trees) with an AUROC of 0.973 ± 0.001 (see 1-Epitope-classifiers-7ML.ipynb).

**Figure 1:**
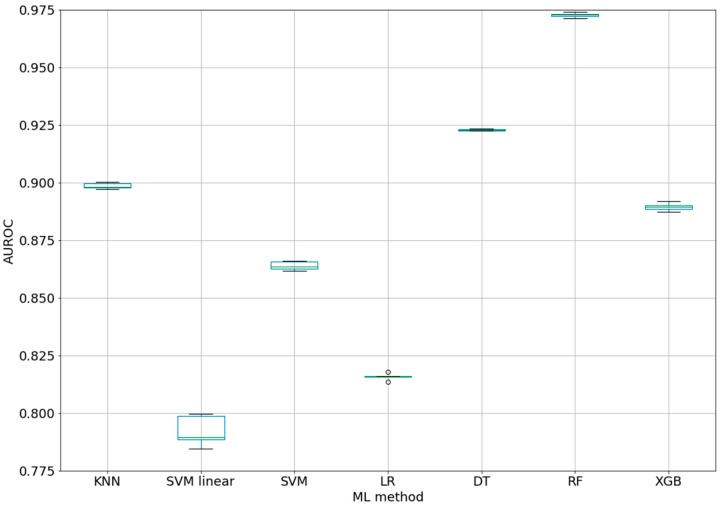
Box-plot for AUROC values of ML classifiers (five-fold CV).

Figure 1 shows the box-plot of the AUROC values for all the ML methods. The AUROC values for the five folds have very small variations (SD between 0.0003 for DT and 0.0067 for SVM linear). This suggests that the AUROCs for all the ML methods are stable within each fold. In addition, the high difference between the RF and the other methods (box-plots are far from overlapping) demonstrated that the results were statistically significant.

**Figure 2:**
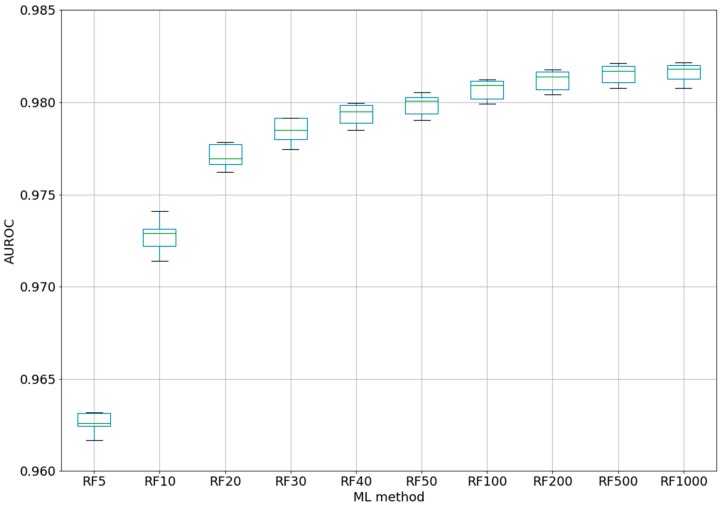
Box-plot for AUROC values of RF classifiers with different trees (five-fold CV). RF*n* = Random Forest with *n* trees (*n* = 5, 10, 20, 30, 40, 50, 100, 200, 500, 1000).

For the best ML method, RF, a test was performed using different numbers of trees, from 5 to 1000 (five-fold CV) (see Figure 2). Even if for the classic QSAR model, RF could reach a constant performance after only 50 trees [25], we tested this assumption with our complex model based on difference between the perturbations of the molecular descriptors under experimental conditions. Even with five trees, the classifier was able to obtain an AUROC of 0.963. After 100 trees, AUROC was higher than 0.98, but no significant improvements were obtained by increasing the number of trees to 200–1000. By increasing the number of trees from 10 to 20, from 20 to 40, or from 40 to 100, statistically significant improvements were obtained for the AUROC values. Therefore, we chose the best models as RF100 (RF with 100 estimators/trees), with an AUROC average of 0.981 ± 0.0005 (see 2-Epitope-RF-trees.ipynb).

## 3. Discussion

The prediction of new epitopes represents a challenge for the vaccine design. In a previous study [16], a linear classifier (linear discriminant analysis (LDA) [26]) was proposed with accuracy, sensitivity, and specificity between 71 and 80% (training and test subsets). The classifier was able to predict the epitope activity of a query peptide under a set of experimental conditions and using a reference peptide. Therefore, the input features consisted of peptide molecular descriptors calculated with S2SNet software and the derived features that mixed original peptide descriptors with experimental data applying the perturbation theory. The study published the dataset containing 1,048,190 pairs of query and reference peptide sequences. The dataset was based on 83,683 peptides sequences, 1448 epitope organisms, 323 host organisms, 15 types of in vivo processes, 28 experimental techniques, and 505 adjuvant additives. The model demonstrated the power of QSAR models using peptide descriptors and the perturbation theory. 

The proposed linear classifier has several limitations. The relationships between the molecular properties and their activity are not always linear. In fact, most of the relationships in nature are not linear. This could explain the maximum performance of only 80% (accuracy). The non-linear Machine Learning methods could offer non-linear relationships between the molecular properties and their activity/property. This is the main reason of the current study, where several non-linear methods were tested. Therefore, seven ML methods were used, including KNN, linear SVM, non-linear SVM using radial basis function (RBF) kernels, LR, and tree-based methods, such as DT, RF, and XGB.

Due to an unbalanced dataset (the number of instances is different in the classes), the accuracy metrics was not the most accurate. Consequently, there was a need for better metrics, such as AUROC and the use of class weighting in ML training. In addition, a single split of data did not provide the best statistical results. Thus, all the current calculations used five-fold CV. The published dataset was corrected by eliminating the duplicate instances too. The availability of an open repository with the dataset, scripts, and results offer the possibility to reproduce all the results just by executing these scripts. 

The statistical significance is very important in ML, and boxplots were therefore created with the scripts in order to present the distribution of the AUROC values with each fold (split of dataset in training and test subset). It was therefore possible to check the spread of the results, the median value, and the outlier values. Thus, we were able to choose random forest as the best ML method. In addition, we checked how the number of trees/estimators in RF could influence the AUROC values. The results demonstrated limitations in increasing the number of trees to 100. From this value (0.981), the gain in AUROC was not statistically significant and the computational effort was not proportional.

**Figure 3:**
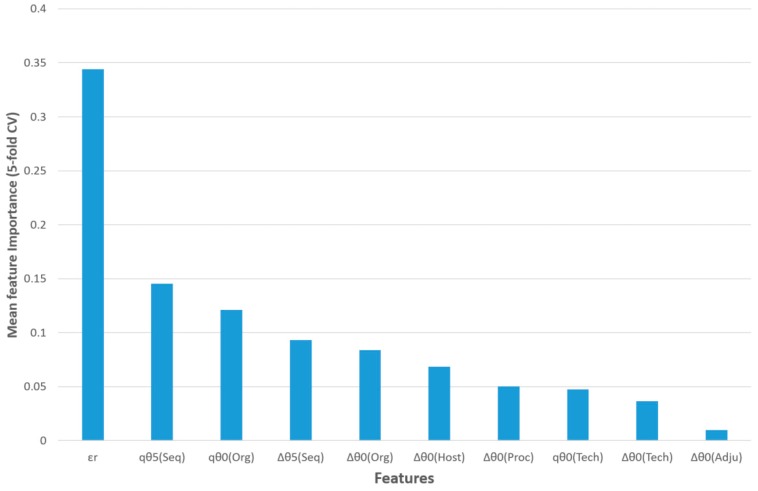
Feature importance for the best RF classifier.

The feature importance of this model for five-fold CV is shown in Figure 3. The query epitope activity class (output variable) was predicted using the observed activity of a reference epitope, three query epitope perturbations of S2SNet descriptors, and six differences between the perturbations of the S2SNet descriptors of query and the reference epitope sequence (under the same experimental conditions). Thus, the observed reference epitope activity (*ε*_r_) was the most important input feature, followed by the perturbation of the Shannon entropies of the query epitope sequence for *k* = 5 and *k* = 0 in the sequences and organisms, ^q^θ_5_(Seq) and ^q^θ_0_(Org). The next two features referred to differences between perturbations in the sequences and organisms: ∆θ_5_(Seq), ∆θ_0_(Org). It was observed that, for the epitope activity, the most important information was encoded into the sequence and depended on the organism. On the contrary, the difference between the perturbations of Shannon entropies for query and reference epitopes in the same adjuvant additives conditions (∆θ_0_(Adju)) was less important for this model.

In conclusion, the current study improved the previous linear classifier using a non-linear classifier (RF), better metrics, such as AUROC, statistical significance using five-fold cross-validation, and offering the script for the reproducibility of the results. This methodology could be used to improve the in silico screening of peptides for a new epitope activity.

## 4. Materials and Methods 

The current work improved the classifier for epitope prediction using a dataset published in a previous paper [16]. The dataset contains information about the linear B-cell epitopes reported in the IEDB database (http://www.iedb.org): 83,683 peptides sequences, 1448 epitope organisms, 323 host organisms, 15 types of in vivo processes, 28 experimental techniques, and 505 adjuvant additives. The features are based on S2SNet peptide descriptors, query and reference peptide sequences and experimental conditions. Using the perturbation theory, the transformed descriptors were calculated (see Ref. [16]). Thus, the proposed classifiers are QSAR models (peptide structure – activity relationships) using perturbations of the molecular descriptors.

The current study presented a state-of-the-art classifier for in silico B-cell epitope prediction before its synthesis in order to save time and money in vaccine design. The classifier represents a Quantitative Structure-Activity Relationship (QSAR) [27] model obtained with Machine Learning methods. The structure of query and reference peptide sequences were transformed into descriptors of sequence recurrence networks using S2SNet software [28]. The initial descriptors were transformed into perturbation descriptors using the perturbation theory for specific experimental conditions. The dataset was available from Ref [16].

The dataset presented in the previous work [16] was used to evaluate the level of epitope activity as a class (query sequence). This output was assessed using information about a pair of epitope sequences: query and references epitopes. The final features of the models are: the observed activity of a reference epitope, the perturbations of Shannon entropies of the query epitope Star graphs under specific experimental conditions, and the differences between these perturbations for query and reference epitopes in the same conditions. Thus, the prediction of a new epitope activity (query epitope) should use an already known activity of a reference epitope and perturbations of graph-type molecular descriptors of both epitope sequences.

**Figure 4:**
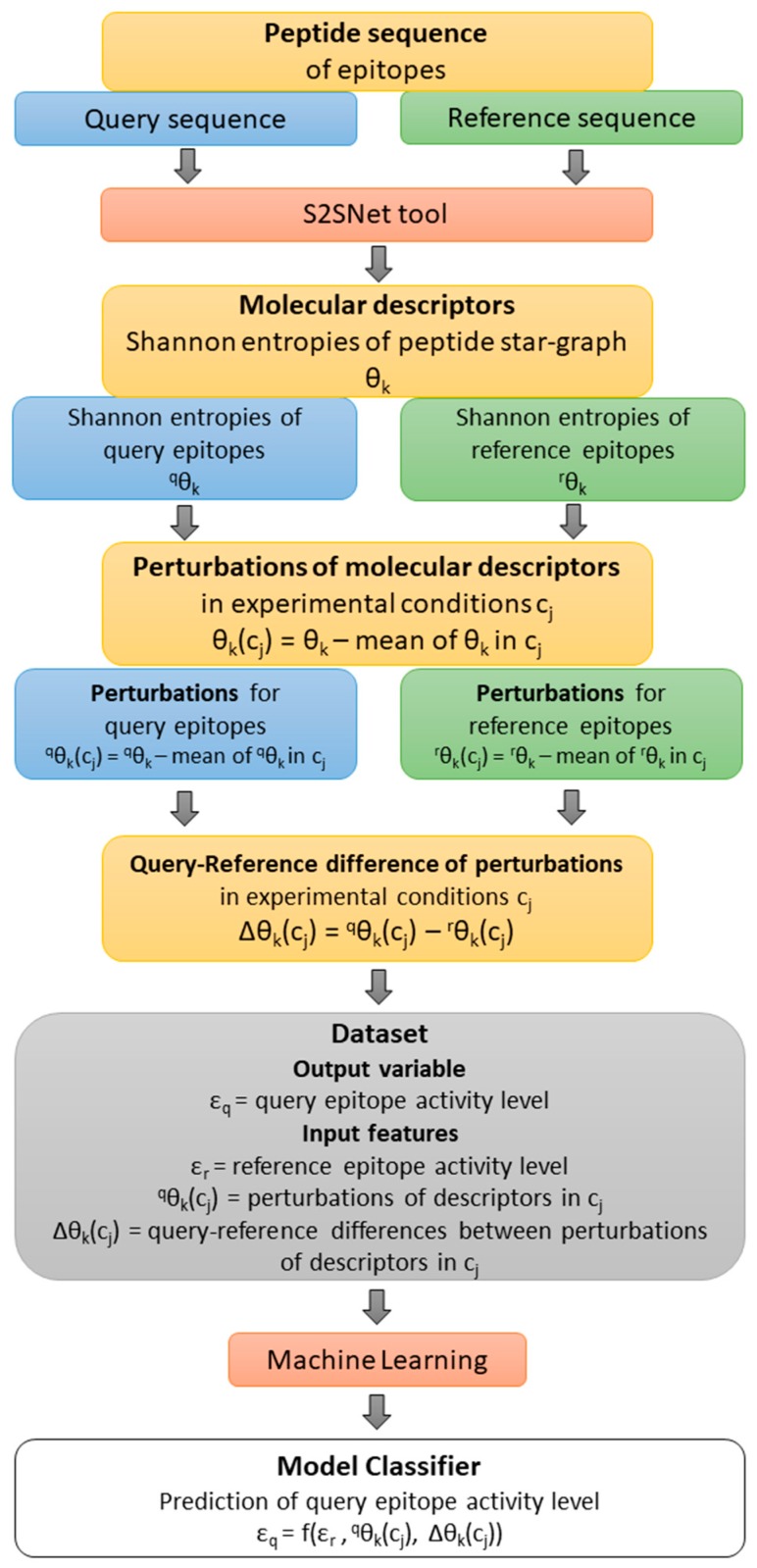
Methodology flow for building models to predict epitope activity level.

The 10 input features are *ε*_r_, ^q^θ_5_(Seq), ^q^θ_0_(Org), ^q^θ_0_(Tech), ∆θ_5_(Seq), ∆θ_0_(Host), ∆θ_0_(Adju), ∆θ_0_(Proc), ∆θ_0_(Org), and ∆θ_0_(Tech). Figure 4 shows a flow of the methodology: from the query-reference epitope sequences, the molecular descriptors and their perturbations under experimental conditions were calculated. *ε*_r_ is the observed value of the epitope activity for the reference (r) peptide sequence. The Shannon entropy information measures for query (q) and reference (r) sequences are ^q^θ_k_ and ^r^θ_k_ (*k* = natural powers of the Markov matrix used in the S2SNet software). ^q^θ_k_(c_j_) represents the perturbations of ^q^θ_k_ for c_j_ factor/experimental conditions such as peptides sequences (Seq), epitope organisms (Org), host organisms (Host), in vivo processes (Proc), experimental techniques (Tech), and adjuvant additives (Adj). Thus, ^q^θ_5_(Seq), ^q^θ_0_(Org), ^q^θ_0_(Tech) represent the perturbations of peptide Shannon entropies for query sequence with Seq, Org and Tech. Δθ_k_(c_j_) represents the differences between the perturbations of Shannon entropies for query and reference peptides for c_j_ factors/experimental conditions ^q^θ_k_(c_j_) – ^r^θ_k_(c_j_). Thus, ∆θ_5_(Seq), ∆θ_0_(Host), ∆θ_0_(Adju), ∆θ_0_(Proc), ∆θ_0_(Org), and ∆θ_0_(Tech) are the corresponding differences of perturbations for Seq, Host, Adj, Proc, and Org. For details, please check Ref. [16]

Due to the classification task, the epitopes were grouped into two classes according to the intensity of the immunogenic response related to this epitope (*ε*). The resulting classes are positive-high epitopes (*ε* = 1) and positive-intermediate epitopes (*ε* = 0). Since the biological assays were used to determine the intensity of the immunogenic response under different experimental conditions cj, *ε* was described as a function of cj. ^q^θ_k_(c_j_) / ^r^θ_k_(c_j_) are perturbations of ^q^θ_k_ / ^r^θ_k_ for c_j_ factor/experimental conditions such as 83,683 peptides sequences (Seq), 1448 epitope organisms (Org), 323 host organisms (Host), 15 types of in vivo processes (Proc), 28 experimental techniques (Tech), and 505 adjuvant additives (Adj). Δθ_k_(c_j_) are the differences between the perturbations of Shannon entropies for query and reference peptides for c_j_ factors/experimental conditions (^q^θ_k_(c_j_) – ^r^θ_k_(c_j_)). After duplicate removal (dataset preprocessing), the dataset contains 709,100 instances and 10 features.

Given that the corrected dataset was slightly unbalanced and we used different ML methods, there was a need of class weighting for the Machine Learning classifiers (Class 0: 0.77, Class 1: 1.42 or Class 0: 1.00, Class 1: 1.84). Seven Machine Learning methods were used: k-nearest neighbors algorithm (KNN) [19], support vector machine (SVM linear and SVM non-linear based on radial basis functions, RBF) [20], logistics regression (LR) [21], decision tree (DT) [22], random forest (RF) [23], and XGBoost—an optimized distributed gradient boosting library (XGB) [24]. A more detailed explanation about the design methodology can be found in the previous works of the authors [29,30]. All the calculations used python/sklearn with Jupyter notebooks. The pipelines contain random stratified five-fold splits (specific to unbalanced datasets to maintain the same ratio of instances by class in the splits) for an outer five-fold cross-validation. Five-fold CV means that for each fold, 80% of the dataset was the training subset and 20% the test subset. For the same reason of the unbalanced classes, the accuracy metrics was removed with the Area Under the Receiver Operating Characteristics (AUROC) [17]. The project offers the possibility of reproducing the results by making available all the scripts, dataset, and results at https://gitlab.com/muntisa/machine-learning-for-peptide-epitopes/.

KNN is one of the most commonly known non-parametric classifiers in the ML field, which assigns an unclassified sample to the same class as the nearest of k samples in the training set [19]. This project uses *k* = 5. In SVM, the input data is non-linearly mapped to a higher dimensionality space, where a linear decision surface can be established [20] using Gaussian radial basis (RBF) kernel functions. LR [21] represents a linear model able to estimate the probability of a binary response using different factors.

DT is a set of decision rules inferred from the features into a tree structure rules (the paths from root to leaf represent classification rules) [22]. RF represents its aggregating decision trees (in parallel) [23]. Thus, RF was characterized by low-bias, low correlation between individual trees, and high variance. Another tree-based ensemble method is XGB—sequential trees [31] with weak classifiers to correct errors.

In the first step, the AUROC of the seven ML methods were statistically tested using the boxplots of the AUROC values for all folds. In the second step, the best ML classifier was tested for different hyperparameters (ex: for RF—number of trees/estimators). The scripts automatically calculated all the results and plotted the boxplots.

## Figures and Tables

**Table 1:** Area Under the Receiver Operating Characteristics (AUROC) values for seven Machine Learning (ML) methods (five-fold cross-validation (CV)).

ML	Fold 1	Fold 2	Fold 3	Fold 4	Fold 5	Mean	SD
KNN	0.900	0.898	0.900	0.898	0.897	0.899	0.0013
SVM linear	0.785	0.800	0.789	0.799	0.790	0.792	0.0067
SVM	0.866	0.863	0.864	0.866	0.862	0.864	0.0019
LR	0.818	0.816	0.816	0.816	0.814	0.816	0.0015
DT	0.923	0.923	0.923	0.923	0.923	0.923	0.0003
RF	0.974	0.973	0.973	0.972	0.971	**0.973**	**0.0010**
XGB	0.892	0.890	0.890	0.889	0.887	0.890	0.0017

ML = Machine Learning; SD = standard deviation; KNN = KNeighborsClassifier, SVM linear = SVC (kernel=“linear”), SVM = SVC (kernel=“rbf”), LR = LogisticRegression, DT = DecisionTreeClassifier, RF = RandomForestClassifier, XGB = XGBClassifier; the best AUROC value and the corresponding SD are bolded.

## Abbreviations

ML	Machine Learning
QSAR	Quantitative Structure-Activity Relationship
MHC	Major histocompatibility
IEDB	Immune Epitope Database
CV	Cross-validation
S2SNet	Sequences to Star Networks software
LDA	Linear discriminant analysis
KNN	k-nearest neighbors algorithm
SVM	Support vector machine
RBF	Radial basis functions
LR	Logistics Regression
DT	Decision tree
RF	Random forest
XGB	Optimized distributed gradient boosting
AUROC	Area Under the Receiver Operating Characteristics
